# A feasibility, acceptability and fidelity study of a multifaceted behaviour change intervention targeting free-living physical activity and sedentary behaviour in community dwelling adult stroke survivors

**DOI:** 10.1186/s40814-020-00603-3

**Published:** 2020-04-29

**Authors:** Sarah A. Moore, Leah Avery, Christopher I. M. Price, Darren Flynn

**Affiliations:** 1 Stroke Research Group, Institute of Neuroscience Newcastle University, 3-4 Claremont Terrace, Newcastle upon Tyne, UK; 2 Stroke Northumbria, Northumbria Healthcare NHS Foundation Trust, Rake Lane, North Shields, Tyne and Wear, NE29 8NH UK; 3 Centre for Rehabilitation, Exercise & Sports Science, School of Health & Social Care, Teesside University, Middlesbrough, TS1 3BX UK

**Keywords:** Stroke, Physical activity, Sedentary behaviour, Behaviour change, Feasibility, Acceptability, Fidelity

## Abstract

**Background:**

Despite the benefits of physical activity for walking ability, balance, and mood, less than 30% of stroke survivors engage in recommended levels of physical activity with high levels of sedentary behaviour observed. This study aims to assess the feasibility, acceptability and fidelity of a theory- and evidence-based multifaceted behaviour change intervention targeting free-living physical activity and sedentary behaviour after stroke.

**Methods:**

This study will be set in community stroke services in the North East of England and will assess the feasibility of a behaviour change intervention targeting free-living physical activity and sedentary behaviour of stroke survivors *and* consultation behaviour of the healthcare professionals to support stroke survivors to make these lifestyle changes. Up to 35 stroke survivors currently receiving stroke rehabilitation within the study catchment area with capacity *and* no contraindications to increasing physical activity/reducing sedentary behaviour will be recruited. Stroke survivors will receive a supported self-management physical activity/sedentary behaviour programme incorporating provision of information, goal setting, action planning, barrier identification, coping planning, self-monitoring and feedback on physical activity and sedentary behaviour. The programme will be supported by up to 12 healthcare professionals (HCPs) recruited from the community stroke services taking part in the study. The HCPs will deliver at least two face-to-face sessions (baseline, review and subsequent reviews if necessary) and provide a range of personalised tools to support each individual stroke survivor (e.g. workbook, self-monitoring tools, information on local resources). The consultation behaviour of the HCPs will be targeted via a training programme incorporating face-to-face training, a training manual and individual feedback on intervention programme delivery from the study research team. The feasibility, acceptability and fidelity of the study protocol will be assessed.

**Discussion:**

The most effective methods of supporting stroke survivors to alter physical activity and sedentary behaviour have yet to be established. This study will establish the feasibility of delivering a complex theory- and evidence-based intervention targeting the behaviour of both stroke survivors and HCPs and assess whether it is acceptable to the target populations. Findings will inform the iterative development of the intervention before a larger scale evaluation.

**Trial registration:**

Trial register: Trial identifier: ISRCTN35516780, date of registration: 24/10/2018

## Background

Increasing physical activity levels and reducing sedentary behaviour (e.g. the time spent sitting or lying) can improve health outcomes and reduce mortality [1, 2]. Home and community physical activity levels are consistently low after stroke, with stroke survivors spending the majority of their day either sitting or lying down [3, 4]. Numerous barriers to engagement in physical activity have been cited after stroke including stroke-related impairments, lack of professional support, reduced self-efficacy and cost and access to facilities [5, 6]. These complex barriers may account for the low levels of physical activity observed after stroke and explain why at present the most effective methods of engaging stroke survivors in long-term free-living physical activity and reducing sedentary behaviour have yet to be ascertained.

Structured exercise is one method of increasing physical activity and has been shown to lead to short-term improvements in walking performance [7], physical fitness [8], metabolic risk factors [9] and mood and quality of life [10]. These short-term benefits however do not appear to be sustained, and the predominant mode of delivery of structured exercise (face-to-face group work) presents with numerous barriers to engagement including access, cost, sustainability and transport. Arguably, the lack of long-term benefit of structured exercise is due to a lack of emphasis on ‘free-living’ behaviour and self-management during this mode of delivery. This argument is supported by previous research findings indicating structured exercise sessions may only improve free-living physical activity levels if delivered alongside tailored counselling (e.g. group/individual counselling strategies including goal setting, monitoring and review) [11]. Interventions that promote supported self-management, e.g. where individuals are provided with the information and skills to effectively manage their long-term condition, can improve long-term outcomes after stroke [12]. Supported self-management may therefore be a useful mode of delivery for an intervention promoting physical activity and sedentary behaviour change after stroke. A number of small feasibility studies have indeed demonstrated that supported self-management is feasible for targeting physical activity/sedentary behaviour after stroke [13, 14].

It is argued that interventions that target behaviour change should have a strong theoretical background to promote change and increase understanding of the determinants of that change. Although complex interventions, few of the existing physical activities and other lifestyle interventions tested post-stroke are based on theory or developed using frameworks [15, 16], which may be why minimal effects have been observed. Interventions based on behaviour change theory in other long-term conditions such as rheumatoid arthritis and lower back pain have shown improvements in long-term physical activity [17, 18]. There is a dearth, however, of research of this type in stroke. Use of theory and frameworks to develop interventions to target physical activity and sedentary behaviour after stroke may lead to an increased likelihood of improving long-term behaviour.

### Intervention development

#### Stroke survivor component

Results of a systematic review and in-depth qualitative study conducted by our study team, and usability/acceptability testing of prototypes of components of the intervention, have been used to develop the content of the stroke survivor intervention.

We have conducted a systematic review of the literature on interventions targeting long-term physical activity and sedentary behaviour following stroke [19]. Intervention study components were established using the Template for Intervention Description and Replication (TIDieR) framework [20] and the Behaviour Change Taxonomy V1 [21]. Nine interventions targeting physical activity were identified by the review, and six of those were rated as very/quite promising. No interventions were identified targeting sedentary behaviour. Face-to-face and telephone supervised support were rated as promising modes of delivery. Intensity of support provided did not alter intervention promise. Behaviour change techniques (BCTs) identified as promising were action planning, goal setting behaviour, problem solving, biofeedback, feedback on behaviour, credible source, social support, information about health consequences and information about social-environmental consequences. Only two of the interventions identified from the included studies were theory-based.

Alongside the review, an in-depth qualitative study has been conducted [22]. The qualitative study identified the barriers and enablers to participation in physical activity and reducing sedentary behaviour after stroke from the perspective of stroke survivors, informal carers and healthcare professionals (see healthcare professional intervention development below). The findings were thematically analysed using the Theoretical Domains Framework (TDF) [23], and this framework was used to map emergent behaviour change techniques and theory related to the behaviour of the stroke survivors. Five major themes were identified that were linked to both consultation behaviour of healthcare professionals supporting stroke survivors to increase physical activity and reduce sedentary behaviour after stroke and the views identified by the stroke survivors and informal carers. These themes were education/training, information provision, daily activities, resources and confidence.

Findings of the systematic review and the qualitative study, alongside an assessment of the community team’s resources, were combined to provisionally map the content of the current stroke survivor intervention, in terms of theoretical domains and constructs, behaviour change techniques and intervention components. Following this provisional mapping exercise, feedback on acceptability and usability of the intervention and intervention tools was gathered from a series of workshops/interviews/questionnaires with stroke survivor and informal carers and healthcare professionals (HCPs). The intervention components were iteratively developed based upon this feedback to finalise the intervention for this study. The development of the intervention will be described in full in a separate publication.

#### Healthcare professional training (HCP) programme

The content of the HCP training programme has been developed in part from our qualitative study where we investigated the perceived barriers and enablers to HCPs supporting people with stroke to increase physical activity and reduce sedentary behaviour. The study identified the following BCTs likely to target the behaviour of the HCPs: problem solving, action planning, monitoring of outcomes of behaviour without feedback, social support (practical), social support (emotional), instruction on how to perform a behaviour, information about health consequences, salience of consequences, demonstration of behaviour and credible source. These BCTs have been incorporated into the HCP training programme which is underpinned by Social Cognitive Theory [24]. The training programme was piloted on a group of HCPs who will not be involved in the study to determine appropriate content and mode of delivery.

### Aim

The overall aim of this study is to assess the feasibility, acceptability and fidelity of a theory- and evidence-based multifaceted behaviour change intervention targeting free-living physical activity and sedentary behaviour after stroke.

#### Objectives

1. To determine stroke survivor and HCP study recruitment and attrition rates
2. To report feasibility of intervention delivery (mode, delivery time, application of intervention components, safety)
3. To report completeness of baseline and review descriptive data to determine the feasibility of the inclusion criteria and of potential outcome measures
4. To obtain views of stroke survivors on the acceptability of the intervention and study protocol
5. To assess the fidelity of delivery, receipt and enactment of the intervention
6. To assess the reaction, attitudes and skills of the HCPs pre and post training programme
7. To assess the cognitive and behavioural skills of the HCPs in response to training programme

## Methods

### Study setting

The study will be set in NHS community stroke services in the North East of England (a list of study sites is available at http://www.isrctn.com/ISRCTN35516780).

### Study design

This is a multi-centre feasibility and acceptability study. The multifaceted behaviour change intervention will target both the free-living physical activity and sedentary behaviour of stroke survivors, and the consultation behaviour of the healthcare professionals supporting stroke survivors to target these behaviours.

#### Progression criteria

Progression criteria to the next stage of the study have been developed in accordance to the traffic light system described by Avery et al. [25]:

Green—Progression to the next stage of the study is appropriate without alteration to the protocol

Amber—Progression to the next stage of the study is appropriate with iterative changes to the protocol

Red—Progression to the next stage of the study is not appropriate

Progression criteria:

- Green—The proposed sample size is achieved and retained, the intervention is considered acceptable and feasible by healthcare professionals, stroke survivors with only minor changes are required (e.g. minor changes to written information content), the study procedures are considered acceptable and feasible and at least 85% of outcome data is collected (i.e. case report form and questionnaire completion).
- Amber—At least 14 stroke survivors (based upon recommendations made by Julios et al. [38]) and at least 7 healthcare professionals were recruited and retained at follow-up, moderate changes to the intervention are required (e.g. additional time required to deliver review sessions or additional intervention content is required), moderate changes to the protocol are required (e.g. amendments to recruitment strategy and data collection procedures) and a minimum of 60% of outcome data is collected.
- Red—< 14 stroke survivors and < 7 healthcare professionals were recruited and retained at follow-up, significant changes to the intervention are required (e.g. change in mode of delivery, frequency of sessions and content of intervention), significant changes to the protocol are required (e.g. eligibility criteria in order to achieve sample size, alternative data collection tools/instruments) and < 60% of outcome data was collected.

#### Stroke survivor eligibility criteria

Inclusion criteria:

- Adult community dwelling stroke survivors
- Currently receiving outpatient/community stroke rehabilitation in the study catchment area
- Capacity to provide informed consent and capability to undertake a supported self-management physical activity intervention programme (e.g. not requiring high levels of hands on therapy for function).
- Agreement by the therapist and patient that there is capacity for increased activity / less sedentary behaviour

Exclusion criteria:

- Contraindications for undertaking physical activity e.g. presence of unstable heart disease
- Advised by GP/Consultant not to undertake physical activity for health reasons

#### Healthcare professional eligibility criteria

- Currently working as a HCP (e.g. physiotherapists, occupational therapists) delivering home/community based stroke rehabilitation
- Capacity and willingness to undertake intervention training, delivery and assessment throughout study duration
- Ability to deliver the intervention programme to up to five stroke survivors

### Sample size

We aim to recruit and retain up to 35 stroke survivors (in line with standard guidance for feasibility studies [26]). As this is a feasibility study, a formal sample size calculation has not been undertaken. The sample size has been selected to allow adequate testing of the intervention and has been based on local resources. We aim to recruit up to 12 HCPs to take part in the training programme and intervention delivery. This sample size has been decided upon based on feedback from study sites.

### Case ascertainment recruitment and consent

#### Stroke survivors

Eligible stroke survivors will be identified by a HCP or a clinical trials officer (CTO) who will explain the nature of the study and provide the stroke survivor/informal carer with an information sheet. After allowing sufficient time for this information to be considered, and an opportunity to ask questions, informed written consent will be obtained from the stroke survivor. Where a stroke survivor is able to provide consent but is unable to sign the consent form (e.g. because of weakness of the dominant hand following stroke), consent will be confirmed orally in the presence of a witness (an individual not otherwise involved in the study) who will sign the consent form on behalf of the stroke survivor.

The original consent forms will be retained at the study sites, and a copy will be given to the stroke survivors. A copy of each stroke survivors’ consent form will be filed in their medical notes at the study sites.

Due to the nature of this study and its small size, we plan for the information sheet and consent form to be available only in English. However, interpreters and translation of written material will be considered should a potentially eligible stroke survivor require this support to participate.

#### Recruitment strategies

Training will be provided to all individuals who can identify eligible participants for the study. These individuals will be contacted on a regular basis by the study co-ordination team to promote recruitment.

#### Loss of capacity to consent to research during participation in the study

It is possible that the stroke survivors recruited to this study may temporarily (e.g. because of intercurrent illness) or permanently (e.g. because of further stroke) lose the capacity to consent to this research project. In either case, it is unlikely that they will be able to continue to participate in a study which involves self-management. In the event of likely temporary incapacity, the intervention will be stopped whilst the participant is unwell, but restarted on recovery if the participant wishes to continue. In the event of permanent incapacity, the participant will be withdrawn from the study. Data collected prior to withdrawal will be retained and used in the study analysis as documented in the patient information sheet.

#### Healthcare professionals

Eligible healthcare professionals (HCPs) will be identified and approached by the study research team, a discussion about the study will be held, and the HCPs will be provided with an information sheet. After allowing sufficient time for this information to be considered, and an opportunity to ask questions, the study research team will obtain written consent from the HCPs.

The original consent forms will be retained at the study sites, and a copy will be given to the HCPs.

### Data collection

After consent, the following data will be collected to characterise the cohort and inform future studies:

#### Stroke survivors

Age (years); sex (male/female); pre-morbid walking status (independent/walks with an aid); marital status (single, married/remarried, separated, widowed, divorced); occupation pre-stroke (e.g. school teacher, retired); current work status (full-time paid, part-time paid, causal, registered sick/disabled, retired, unemployed, student); medical history (diabetes, arthritis etc.); geographical location (post code) education (year count); date of stroke; assistive device use (no device/stick/tripod/elbow crutches/zimmer frame/delta frame/four wheeled walker/wheelchair/other); Rivermead Mobility Index [27]; Fatigue Assessment Scale [28]; Warwick-Edinburgh Mental Well-being Scale (WEMWBS) [29]

#### HCP demographic information

Place of work, sex, occupation, years qualified, years specialising in stroke and employment status (whole-time equivalent)

### Stroke survivor intervention

#### Procedures

**Fig. 1:**
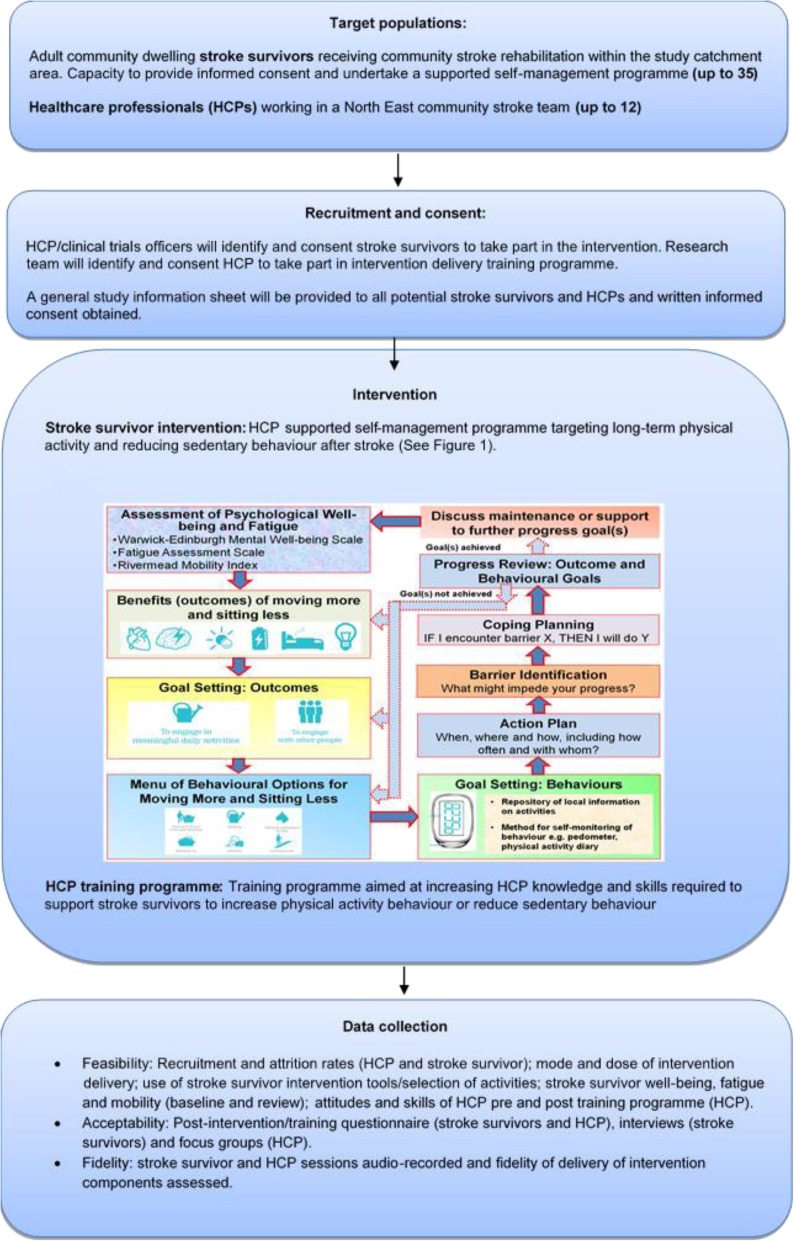
Stroke survivor intervention programme

The stroke survivor intervention will target free-living physical activity and sedentary behaviour through a supported self-management programme. The supported self-management programme is underpinned by the Health Belief Model [30] and Self-Regulation Theory [31]. The following 17 behaviour change techniques (BCTs) have been incorporated into the programme: information about health consequences, salience of consequences, social support (unspecified), instruction on how to perform the behaviour, demonstration of the behaviour, goal setting (behaviour), problem solving, information about antecedents, monitoring of emotional consequences, information about health consequences, information about social and environmental consequences, social reward, habit formation, credible source, action planning, feedback on behaviour and self-monitoring of behaviour. These BCTs are used to target each construct of the behaviour change theories/models and thus aim to operationalise the models in order to target and change the target behaviours. The intervention will be supported by HCPs. The first face-to-face session/s the stroke survivor has with the HCP will consist of assessment of psychological well-being, fatigue and mobility using reliable and valid questionnaires. This will be followed by identification of preferred behavioural outcome, activity selection, personalised goal setting (behaviour and outcomes), action planning, barrier identification and coping planning (Fig. 1). The stroke survivor’s progress will then be reviewed by the HCP. The progress review session/s will be timed around the short-term goals (reviewed within less than three months) identified by the stroke survivors, e.g. if a goal is set for three weeks’ time, the review session will be delivered three weeks from when the goal was set. The review will focus on progress towards outcomes and behavioural goals. If goals have been achieved, maintenance or support to further progress goals will be provided. The overall aim of the intervention is to equip the stroke survivors with the knowledge and skills to enable them to self-manage their physical activity and sedentary behaviour in the long-term.

#### Materials

All stroke survivors will be provided with a paper-based workbook. The workbook contains questionnaires on psychological well-being, fatigue and mobility; information on the benefits/potential outcomes of moving more and sitting less; a list of potential activities, goal setting/action planning/barrier identification/coping planning and progress review sheets. Alongside the workbook, a number of tools will be used on an individual basis including pedometers and instructions, information on apps, physical activity diaries and wall planners and a repository of physical activity resources in the local area. For example, if a stroke survivor would like to increase their daily step count, they may be provided with a pedometer to enable self-monitoring of this activity, or if they wish to break up their sedentary time by standing every hour, they may be advised on a commercially available app that provides feedback on sitting time. The intervention materials that will be used on an individual basis are listed in Table 1 alongside their relationship to the theoretical constructs of the health behaviour change models used to underpin the programme and specific behaviour change techniques.

**Table 1:** Intervention materials and their relationship to theoretical constructs and behaviour change techniques

Intervention programme materials	Form and information content	Theoretical constructs	Behaviour change techniques
Workbook	Paper-based workbook incorporating general advice on moving more and sitting less, self-assessment measures of well-being, fatigue and mobility, benefits of moving more and sitting less, potential outcomes list; physical activity options; goal setting; self-monitoring, action planning; barrier identification, progress review; example goal sheets	Health Belief model (HBM) Constructs: all constructs of HBM Self-regulation theory Constructs targeted: Individual perceptions; likelihood of action; goal setting; problem solving; self-monitoring; feedback	Information about health consequences Salience of consequences Social support (HCP) Instruction on how to perform a behaviour Goal setting Problem solving Information about antecedents Monitoring of emotional consequences Information about emotional consequences Information about social and environmental consequences Habit formation Action planning Feedback on behaviour Self-monitoring
Discussion cards	Laminated discussion cards with potential physical activity options and outcomes	Self-regulation theory Constructs targeted: action planning Health Belief Model Constructs targeted: Individual perceptions	Action planning
Summary sheet	Paper-based sheet with goals, action plans and coping plans	Self-regulation theory Constructs: action planning, goal setting, problem solving	Goal setting (behaviour) Action planning Problem solving
Physical activity self-monitoring tools	Participants will select a physical activity or sedentary behaviour self-monitoring tool from a range of options including: paper-based activity diary; laminated wall activity planner/diary; pedometer; commercially available physical activity monitoring apps	Self-regulation theory Constructs targeted: self-monitoring	Self-monitoring
Physical activity repository	Paper-based repository of locally based physical activity options and online resources	Self-regulation theory Constructs targeted: goal setting	Instruction on how to perform a behaviour Social support

#### Mode of delivery

The self-management intervention programme will be supported by HCPs (e.g. physiotherapists, occupational therapists, rehab assistants) working in community stroke teams. The HCPs will all have specialist knowledge in community stroke rehabilitation. The first session/s will be delivered face-to-face with review sessions delivered either face-to-face or by telephone.

The intervention programme will be initiated once the stroke survivor is moving towards supported self-management and a need to move more and sit less has been highlighted. The time from stroke will vary dependent on needs of the stroke survivor/health care professional’s opinion on the best timing/availability of resources. Participants may still be receiving rehabilitation from the community stroke teams at the same time as the delivery of the intervention.

#### Dose and duration

Stroke survivors will have contact with the trained HCP on at least two occasions. There is no upper limit to the number of HCP supported sessions/time to deliver sessions. The number of sessions will be defined by the stroke survivors’ support needs/availability of resources.

#### Tailoring

The intervention programme will be tailored to support the individual needs of the stroke survivors in relation to ability/preference and values.

### HCP training programme

#### Procedures

The behaviour of the HCPs in supporting stroke survivors to increase free-living physical activity/reduce sedentary behaviour will be targeted by a training programme consisting of face-to-face training with the research team, a training workbook and feedback on the delivery of the intervention.

#### Materials

The HCPs will be provided with a training workbook with activities to aid their development of knowledge and skills to support physical activity/reduce sedentary behaviour after stroke. The HCPs will also be provided with a repository of tools which will be used on an individual basis with stroke survivors (e.g. stroke survivor workbook, pedometers, pedometer instructions, discussion cards, physical activity information repository (see Table 1)).

#### Mode of delivery

The HCP training programme and fidelity assessment will be delivered face-to-face, by telephone and via email contact with members of the research team. The members of the research team who will be providing the training include an HEE/NIHR Clinical Lecturer stroke physiotherapist (qualified in physiotherapy for over 19 years, specialising in stroke for 14 years, specialising in stroke physical activity research for 10 years) and two health psychologists/senior researchers with extensive expertise in the development, evaluation and optimisation of lifestyle behavioural interventions for people with long-term conditions, including delivery of training to HCPs on the deliver behavioural interventions in clinical settings.

#### Dose and duration

The face-to-face training programme will last up to 4 h and be delivered to each community stroke team individually. The training will continue throughout the study through the use of the training workbook and feedback from the research team.

#### Tailoring

The HCP training programme will be tailored to the needs of each individual HCP through feedback on intervention delivery.

### Outcome assessments

#### Stroke survivors

At review sessions, the following outcome assessments will be reassessed: stroke survivor’s psychological well-being (Warwick-Edinburgh Mental Well-Being Scale [29]), fatigue (Fatigue Assessment Scale [28]) and mobility (Rivermead Mobility Index [27]). The number of times these measures are reassessed will be dependent on the number of review sessions they undertake with their healthcare professional.

#### Acceptability of the study intervention

A questionnaire with a combination of closed and open questions will be provided to each of the stroke survivors to gather their views of the intervention programme. Likert scales will be used to explore levels of agreement with different statements on acceptability of different aspects of the intervention, e.g. ‘I had good support from my healthcare professional during my participation in the PARAS study’, Additional comments on each statement and views on any aspect of the intervention that could be changed will allow further exploration of acceptability. Stroke survivors will be provided with a pre-paid envelope to return their questionnaires to the study team.

Alongside capturing the views of the stroke survivors via questionnaire, a sub-sample will be interviewed to determine acceptability of the intervention programme. Face-to-face semi-structured interviews of approximately 1 h will be undertaken. An estimated sample of up to 13 stroke survivors will be interviewed in line with published guidance for theory-based qualitative interview studies. Purposive sampling will be used to recruit stroke survivors who have completed the study. Purposive sampling has been chosen to ensure a maximum variation sample based on gender, age and stroke-related physical disability.

Potential participants will be invited to consent to being contacted to be interviewed during their initial consent to the study. Participants selected for interview will then be contacted either by telephone or by email and given a brief description of what the interview might address, such as how they felt about taking part in the study, engagement with the supported self-management programme and any impact they perceive on everyday activities.

### Healthcare professionals

#### Attitudes skills and reactions to training programme

The attitudes and skills of the HCPs pre- and post-training will be assessed via a questionnaire developed specifically for the study. The questionnaire has been piloted on the HCPs that undertook the pilot testing of the training programme and refined. A separate questionnaire will be used to establish HCP reactions to the training programme.

#### Acceptability of training programme and study protocol

The HCPs will be invited to attend a 2-h focus group at the end of the study to discuss their views on the study protocol. Topics to be covered in the focus group will include the impact/potential benefits of PARAS, feasibility of delivering the stroke survivor self-management programme within usual care, the usability of the intervention tools (e.g. workbook, pedometers and instructions) and the acceptability of the mode and form of the stroke survivor intervention delivery.

#### Cognitive and behavioural skills of the HCPs in response to training programme

Analysis of audio-recordings of face-to-face and review sessions with the HCP will be used to assess the cognitive and behavioural skills of the HCPs delivering the intervention programme. All face-to-face sessions delivered by HCPs with the stroke survivors will be audio recorded. Following published guidance, the content of 20–40% of these recorded sessions will then be analysed [32].

### Fidelity of intervention delivery

#### Fidelity strategies for the design of the study

HCPs involved in the study will have face-to-face training on the study protocol and the components of the stroke survivor intervention programme to standardise the delivery of the intervention. The community stroke team staffing resources will be mapped out before the study to ensure resources are adequate to deliver the intervention programme over the study period. Resource pre-planning will be undertaken in-case HCPs enrolled in the study leave during the period of study delivery.

#### Treatment fidelity strategies for monitoring and improving HCP training

Standardised face-to-face training will be delivered on the study protocol and intervention delivery to all HCPs with direct involvement in the study and to other members of the team who may be supporting the study. During the face-to-face HCP training sessions, the skill acquisition of the HCPs will be assessed to ensure they have adequate knowledge and skills to deliver the intervention. Those who do not have the adequate skills and knowledge will receive additional tailored feedback and training.

#### Treatment fidelity strategies for monitoring and improving delivery of stroke survivor intervention

In order to maximise HCPs’ skills delivering the intervention programme, the first two sessions they deliver with stroke survivors will be audio recorded by the HCPs themselves. The content of these audio-recorded sessions will be analysed by the research team using a fidelity checklist based upon the components of the stroke survivor intervention. Feedback on the delivery of the components of the intervention will then be provided by the research team before the HCPs see more participants. The HCPs will also have email/telephone contact with the research team to request further top-up training.

#### Treatment fidelity strategies for monitoring and improving receipt of stroke survivor intervention

The understanding and cognitive and behavioural skills of the stroke survivors undertaking the intervention programme will be checked by HCPs during face-to-face sessions by asking stroke survivors to independently complete a summary sheet of their goals, action and coping plans after they have completed their workbooks. The content of this summary sheet, use of intervention tools and analysis of audio-recorded sessions will also be used to monitor stroke survivor receipt of the intervention.

#### Treatment fidelity strategies for monitoring and improving enactment of stroke survivor skills

Completion of stroke survivor workbooks and use of intervention programme tools (e.g. physical activity diaries) will be checked at review sessions by HCPs to monitor stroke survivor self-management skills.

### Safety

The safety of the intervention will be evaluated by examining the occurrence of serious adverse events. This study will only report adverse events which are considered to be serious.

The standard definitions for serious adverse events will be used in this study:

*Serious adverse event (SAE)*: an untoward occurrence that:

- Results in death
- Is life-threatening (refers to an event in which the subject was at risk of death at the time of the event; it does not refer to an event which hypothetically might have caused death if it were more severe)
- Requires hospitalisation, or prolongation of existing hospitalisation
- Results in persistent or significant disability or incapacity
- Is otherwise considered medically significant by the investigator

Medical judgement will be exercised in deciding whether an adverse event is serious in other situations. Important medical events that are not immediately life-threatening or do not result in death or hospitalisation but may jeopardise the patient or may require intervention to prevent one of the other outcomes listed in the definition above should also be considered serious.

Serious adverse events *exclude*:

- Pre-planned hospitalisations
- Scheduled treatment for pre-existing conditions.

Capture of potential SAEs will take place at face-to-face review sessions by including the following questions in the outcome pro forma: “are there any new medical problems since the last study visit?” For any events which fulfil the criteria to be a SAE and are unreported, the study SAE form will be completed.

Events considered to be SAEs will subsequently be documented onto a separate study SAE form, and a causality and expectedness assessment will be performed. As study investigators or other members of the research team may become aware of SAEs at times other than at review assessment appointments, the SAE form will also be used to directly capture these events.

Initial/provisional SAE reports can be made by telephone or email to the study co-ordinating centre. All initial/provisional reports must be followed by a fully completed SAE form. If incomplete information is available at the time of this initial report, further information must be provided on a follow-up form as soon as it is available. All SAEs regardless of causality or expectedness will be reported to the Chief Investigator and trial sponsor (Northumbria NHS Foundation Trust) in line with local policies. The main REC will be notified of related and unexpected SAEs within 15 days of the Chief Investigator becoming aware of the event.

### Data analysis

#### Objective 1

Stroke survivor recruitment rate/month (total per site) and attrition rates/site and reasons will be described. HCP recruitment will be described as number of HCPs recruited compared to number of eligible HCPs in each team. Non-completion of training programme (e.g. face-to-face training, workbook, feedback) will be described as a percentage of non-completers out of the total of HCPs recruited.

#### Objective 2

The profession of the HCP delivering the intervention; the number and time taken (minutes) for face-to-face/telephone intervention delivery, the application of the different intervention components applied (e.g. self-monitoring tools, activity selection) and safety reporting will be described.

#### Objective 3

Completeness of outcome measure data collection including questionnaires will be described (number and percentage). Summary descriptive statistics (e.g. mean and standard deviation, median and interquartile range, 95% confidence interval as appropriate) will be used to describe stroke survivor baseline demographics and psychological well-being (Warwick-Edinburgh Mental Well-Being Scale), fatigue (Fatigue Assessment Scale) and mobility (Rivermead Mobility Assessment) at baseline and review.

#### Objective 4

Closed questionnaire data from stroke survivors will be coded and summarised. Open-ended questions have also been included in order to encourage full, meaningful answers using the participants own words that might potentially offer insights or issues not captured in the closed questions, yielding more accurate information to inform future studies. Answers to open questions will be thematically explored in order to identify, analyse and report patterns (themes) within the data.

Thematic analysis has been chosen to analyse the interview data as it is a flexible method that allows themes to emerge from the data [33] and has previously been used to analyse data following exercise interventions [34] and physical rehabilitation following stroke [35]. This rigorous thematic approach can produce an insightful analysis that answers particular research questions [33]. Thematic analysis is one of the most commonly used methods of analysing qualitative data, as it is simple, is less time consuming and has a flexible approach. NVivo software will be used to aid analysis. The data will be transcribed and then repeatedly read and coded by two researchers independently within a framework of a priori issues and those identified by stroke survivors or emerging from the data. Any divergence between coders will be discussed on an on-going basis to inform the analysis and resolve differences in the interpretation of data. Analysis will be discussed at regular meetings of the research team to identify areas for closer consideration (including negative case analysis) and to enhance credibility of the thematic framework and interpretation [32, 36].

#### Objective 5

Delivery, receipt, enactment and adherence to the intervention by stroke survivors and HCP will be analysed from completion of face-to-face baseline and review sessions, case report forms, goal summary sheets and audio recordings of the face-to-face delivery of the intervention. This data will be used to determine the fidelity of the delivery, receipt, enactment and adherence to the intervention.

#### Objective 6

Closed HCP questionnaire data from pre and post the training programme will be coded and summarised. Open-ended questions have also been included in order to encourage full, meaningful answers using the participants own words that might potentially offer insights or issues not captured in the closed questions, yielding more accurate information to inform future studies. Answers to open questions will be thematically explored in order to identify, analyse and report patterns (themes) within the data.

#### Objective 7

Audio recordings of HCP and stroke survivor baseline or review sessions will be transcribed verbatim and used alongside HCP recorded written notes on aspects of intervention delivery. One of the research team trained in the use of the Behaviour Change Taxonomy Version 1 (BCT v1) [21] and an expert coder will independently code all consultation transcripts and written notes to assess skill acquisition (for feedback purposes) and fidelity of delivery of each specific behaviour change technique. A coding frame/fidelity checklist based on the BCT v1 will be used to identify whether each BCT was delivered as planned. Where discrepancies in coding exist, the researcher and expert coder will meet to resolve these via discussion. The percentage of positive agreement will be calculated to assess inter-rater reliability.

## Discussion

The main aim of this feasibility study is to determine the feasibility, acceptability and fidelity of a multifaceted behaviour change intervention targeting free-living physical activity and sedentary behaviour in community dwelling adult stroke survivors. The most effective methods for improving long-term physical activity and reducing sedentary behaviour after stroke have yet to be ascertained. Theory-informed interventions are shown to improve physical activity in other long-term conditions and may be effective post-stroke. The intervention tested within this study has been developed with the use of theory and behavioural mapping, increasing potential for efficacy. The content of stroke rehabilitation interventions is often poorly described [37]. Content of this current intervention has been described using the TIDieR framework [20] which will enable further testing of the components of the intervention and translation of future results. Previous stroke rehabilitation research has been limited by a lack of measurement of fidelity, e.g. whether interventions are delivered as intended. In this study, there is a focus on ensuring and measuring fidelity, which will enable understanding of whether of intervention delivery and inform future knowledge in this field and the iterative development of the intervention to enable future testing.

### General information

#### Study withdrawal

No specific study withdrawal criteria have been set. Participants may withdraw from the study at any time for any reason. Should a HCP or stroke survivor decide to withdraw from the study, a reason for withdrawal will be sought but participants can chose to withdraw without providing an explanation. If a stroke survivor decides to withdraw, it will not affect the normal care they receive either now or in the future. Data collected prior to withdrawal will be used in the study analysis unless consent for this is specifically withdrawn.

Clinical teams, local treatment providers or investigators may also withdraw stroke survivors from the study at any time if they feel it is no longer in the participant’s interest to continue, for example, because of intercurrent illness.

#### Lone worker policy

Study team and intervention providers will follow the relevant Trust and/or Newcastle University lone workers policy when collecting study data and providing therapy in people’s homes.

#### Confidentiality

Personal data will be regarded as strictly confidential. The study will comply with the Data Protection Act, 2018, General Data Protection Regulations and Caldicott Principles. All study records will be kept at study sites and/or Newcastle University with restricted access. All trial documentation will be retained for future audit in line with the sponsor policies. Participants will not be identified in any report or publication arising from this research. Any feedback comments or quotes will be anonymised.

#### Indemnity

NHS Trusts participating in the study have liability for clinical negligence that harms individuals toward whom they have a duty of care. NHS indemnity covers NHS staff and academic staff with honorary contracts conducting the trial for potential liability in respect of negligent harm arising from the conduct of the study. Northumbria Healthcare NHS Foundation Trust is Sponsor, and through the Sponsor, NHS indemnity is provided in respect of potential liability and negligent harm arising from study management. Indemnity in respect of potential liability arising from negligent harm related to study design is provided by NHS schemes for those protocol authors who have their substantive contracts of employment with the NHS and by Newcastle University Insurance schemes for those protocol authors who have their substantive contract of employment with the university. This is a non-commercial study, and there are no arrangements for non-negligent compensation.

#### Data management

A web-based data entry tool will be developed and administered by the research team for the study. A database manager will monitor data quality under supervision of the project team.

### Data sharing

We will share anonymised data (referenced only with study number) with approved collaborators both nationally and internationally (inside and outside of the EU) for scientifically sound, peer-reviewed studies. Data sharing offers a more open approach which allows us to maximise the impact of the study for the health and well-being of the population.

Data sharing will be managed by our data management committee according to the following procedures:

1. Collaborators interested in accessing data from the study will send the data management committee an expression of interest, for example, using data request from or via research platforms data portals.
2. The committee will then review the data request. If required, the data management committee may request changes to the proposed study by collaborators. The data management committee may then approve or reject the proposed study.
3. A data use agreement will be drafted and signed by both parties.
4. As agreed by the data managing committee and collaborators, and according to signed data agreement forms, anonymised data will be transferred to the collaborators.

Data will be securely transferred to collaborators. Data will be securely stored by collaborators for a fixed duration, as stated in the signed data use agreement. Only anonymous and unidentifiable data will be sent.

### Auditing

Progress and quality of trial delivery via fidelity checks will be monitored prospectively by the project management group at scheduled meetings. As this is a feasibility study, it will not be audited by an independent auditing company.

### Dissemination of results

The data will be the property of the Chief Investigator and Co-Investigator(s). Publication will be the responsibility of the Chief Investigator.

The study will be presented at national and international conferences and reported in peer-reviewed journals. Reports will be written for the study sponsor and regulatory bodies. A summary of the results will be sent to study participants. Anonymised data will be provided to research databases as requested (e.g. the Cochrane Collaboration, the Virtual International Stroke Trials Archive (VISTA) to enable future meta-analyses).

## Data Availability

See data sharing section above.

## Abbreviation

BCT: Behaviour change technique
HCPs: Healthcare professionals
SAE: Serious adverse event
TIDieR: Template for intervention description and replication
